# Flow Cytometric Analysis of Bacterial Protein Synthesis: Monitoring Vitality After Water Treatment

**DOI:** 10.3389/fmicb.2021.772651

**Published:** 2021-12-10

**Authors:** Mathilde Lindivat, Gunnar Bratbak, Aud Larsen, Ole-Kristian Hess-Erga, Ingunn Alne Hoell

**Affiliations:** ^1^Faculty of Engineering and Science, Institute of Safety, Chemistry and Biomedical Laboratory Sciences, Western Norway University of Applied Sciences, Haugesund, Norway; ^2^Department of Biological Sciences, University of Bergen, Bergen, Norway; ^3^NORCE Environment, NORCE Norwegian Research Center AS, Bergen, Norway; ^4^Norwegian Institute for Water Research, Bergen, Norway

**Keywords:** flow cytometry, BONCAT, bacteria, water analysis, vitality, UV irradiation, heat treatment

## Abstract

Bacterial vitality after water disinfection treatment was investigated using bio-orthogonal non-canonical amino acid tagging (BONCAT) and flow cytometry (FCM). Protein synthesis activity and DNA integrity (BONCAT–SYBR Green) was monitored in *Escherichia coli* monocultures and in natural marine samples after UV irradiation (from 25 to 200 mJ/cm^2^) and heat treatment (from 15 to 45 min at 55°C). UV irradiation of *E. coli* caused DNA degradation followed by the decrease in protein synthesis within a period of 24 h. Heat treatment affected both DNA integrity and protein synthesis immediately, with an increased effect over time. Results from the BONCAT method were compared with results from well-known methods such as plate counts (focusing on growth) and LIVE/DEAD™ BacLight™ (focusing on membrane permeability). The methods differed somewhat with respect to vitality levels detected in bacteria after the treatments, but the results were complementary and revealed that cells maintained metabolic activity and membrane integrity despite loss of cell division. Similarly, analysis of protein synthesis in marine bacteria with BONCAT displayed residual activity despite inability to grow or reproduce. Background controls (time zero blanks) prepared using different fixatives (formaldehyde, isopropanol, and acetic acid) and several different bacterial strains revealed that the BONCAT protocol still resulted in labeled, i.e., apparently active, cells. The reason for this is unclear and needs further investigation to be understood. Our results show that BONCAT and FCM can detect, enumerate, and differentiate bacterial cells after physical water treatments such as UV irradiation and heating. The method is reliable to enumerate and explore vitality of single cells, and a great advantage with BONCAT is that all proteins synthesized within cells are analyzed, compared to assays targeting specific elements such as enzyme activity.

## Introduction

Determining bacterial physiological states is important for a wide range of applications. Hereafter, these physiological states are described as viable cells when bacteria have the capacity to reproduce (i.e., divide) and vital when they present metabolic activity and cell integrity but are unable to reproduce. Bacterial activity includes reactions, processes, and functions necessary for bacterial growth and development. Investigations into viability and metabolic activity of microorganisms are necessary to assess their role in aquatic ecosystems (Czechowska et al., 2008). For industries that use bacteria in their production, bioprocess monitoring is important to control and maintain optimal conditions throughout the production (Rieseberg et al., 2001). In health research, the determination of effects of antimicrobial treatment is highly relevant to fight resistant bacterial pathogens (Léonard et al., 2016). Analysis of viable microbes in water is necessary to ensure safety and the absence of pathogens. Wastewater, drinking water, aquaculture, or ballast water onboard ships are all the examples where water treatment is required to prevent the spread of pathogens or non-indigenous species.

With such a broad spectrum of applications, a wide range of methods targeting different cellular processes has been developed during the last decades and applied to assess the physiological state and vitality of bacteria. Many of these are based on the use of fluorescent probes including the detection of DNA with permeable stains such as SYTO 9 and SYBR Green I; the control of membrane integrity with impermeable DNA stain propidium iodide (PI); analysis of membrane potential with the passage of charged molecules rhodamine 123 and DiOCx(x) derivate; efflux pump activity with the passive diffusion of small molecules such as ethidium bromide; and respiration activity and the electron chain transportation efficiency with cleavage of substrates INT [2-(p-iodophenyl)-3-(p-nitrophenyl)-5-phenyltetrazolium chloride] and CTC (5-cyano-2,3-ditolyl tetrazolium chloride) (Buysschaert et al., 2016). While developed for fluorescent microscopy, many of these methods have been adapted for flow cytometry (FCM) which often is considered both faster and cheaper (Hoefel et al., 2003). Interpreting staining data is, however, inherently difficult especially when methods are applied to analyze mixed and diverse natural communities (Shi et al., 2007; Hammes et al., 2011; Braissant et al., 2020).

Disinfection methods such as chlorination, ozonation, filtration, and UV irradiation, sometimes in combination, are used to inactivate bacteria (Pichel et al., 2019). To evaluate water disinfection and for compliance control, water microbial quality assessment is required to ensure that cells are removed, inactivated, or dead after treatment. Common methods include culturing and selective plating of pathogens (Allen et al., 2004), microscopy analysis (Dufour et al., 2003), detection of ATP (Lee et al., 2001), and a variety of PCR-based detection of specific pathogens (Ramírez-Castillo et al., 2015). FCM has also been shown to be an efficient instrument to analyze vitality of aquatic microbes (Hammes and Egli, 2010; Hoell et al., 2017; Safford and Bischel, 2019) and has been used for example to evaluate microbial counts in recirculating aquaculture farm (Rojas Tirado et al., 2018). In spite of this, FCM is rarely used as a standard method for water analysis (Safford and Bischel, 2019), perhaps due to the lack of multicolor-based method that can explore different cell parameters at the same time (Hammes and Egli, 2010).

Despite these aforementioned technological advances, the need remains for the development and improvement of methods to measure bacterial vitality and to evaluate water treatment strategies. Bio-orthogonal amino acid tagging (BONCAT) is promising in this respect and has been used for microbial analysis (Hatzenpichler and Orphan, 2015) and for routine analysis of natural marine microbial communities with FCM (Lindivat et al., 2020).

The principle is that alkyne- or azide-bearing amino acid analogs are incorporated during protein synthesis and subsequently labeled with a fluorescent dye by an azide–alkyne click chemistry reaction. BONCAT is efficient for determining bacterial vitality at the community level, but has, to our knowledge, never been used to assess the effect of physical or chemical disinfection procedures (such as UV, temperature, and H_2_O_2_) on bacterial activity. Since various treatments provoke changes in the bacterial metabolism to prevent death, determining whether BONCAT can be used as a reliable vitality indicator is necessary.

In this study, we assess the use of BONCAT in combination with FCM (BONCAT-FCM) as a method for evaluating different water disinfection treatments. The method was applied to monitor cell death and to determine vitality states (live, dead, damaged) in *Escherichia coli* monocultures and compared to plate counts and LIVE/DEAD™ BacLight™ staining. The vitality of bacteria from marine water samples after UV and heat treatments was also assessed with BONCAT-FCM.

## Materials and Methods

### Bacterial Culture Maintenance and Environmental Samples

Monocultures of *E. coli* (ATCC 25922), *Aeromonas salmonicida* (ATCC 33658), *Listonella anguillarum* (ATCC 19264), *Yersinia ruckeri* (ATCC 29473), and *Bacillus cereus* (GMB 105.1, isolated from soil, Norway, provided by the University of Bergen, Department of Biosciences) were grown from frozen stocks on tryptic soy agar (BD Bioscience, United States) at 37, 20, 18, 26, and 30°C, respectively. For natural bacterial communities, surface seawater (<2 m) was collected with a sampling bottle in Haugesund harbor (Haugesund, Norway) and filtered with a 100-μm cell strainer filter (BD Bioscience, United States) to remove larger particles or organisms that could block the fluidic system of the flow cytometer.

### Bacterial Disinfection: Experimental Setup

**FIGURE 1:**
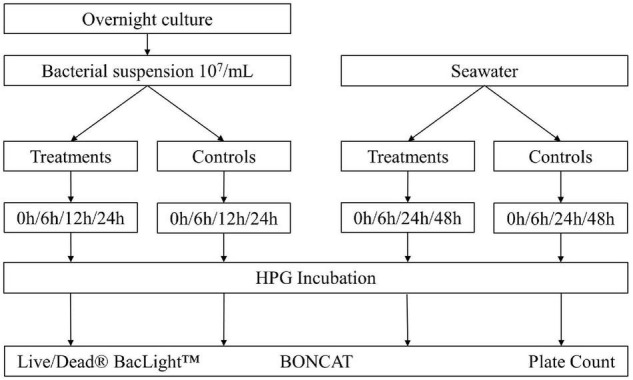
Overview of the experimental setup. *Escherichia coli* was diluted in 1x PBS to a final concentration of 10^6^–10^7^ bacteria/ml. Seawater was directly analyzed after 100 μm prefiltering. Different treatments were applied: UV irradiation with UV doses of 25, 50, 75, 100, and 200 mJ/cm^2^; heat treatment at 55°C for 15, 30, and 45 min. Controls without treatment were incubated with HPG (positive control) and without HPG (negative control). Dead controls with fixed cells (DF) and heat inactivation (3 min at 80°C) (DT) were added for comparison. Samples were analyzed with plate counts, LIVE/DEAD^®^ BacLight™ staining with PI/SYTO9, and BONCAT.

To evaluate the use of BONCAT for assessing the vitality state of bacteria in monocultures and natural aquatic samples, two common bacterial disinfection methods (UV irradiation and heat inactivation) were applied. The BONCAT results were compared with other vitality analysis methods [i.e., LIVE/DEAD™ BacLight™ (Invitrogen, Thermo Fisher Scientific, United States) and plate counts], and the experimental setup in Figure 1 gives an overview of the different steps. Experiments were carried out three times with individual samples in triplicates.

A 10 ml of fresh liquid *E. coli* culture grown overnight (OD ∼1.0) was harvested by centrifugation at 16,000 × *g* (Multifuge 3SR+, Thermo Fisher Scientific, United States) for 5 min at room temperature (RT). The cells were washed 3 times with 1x PBS by centrifugation and resuspension. The bacterial concentration was determined with SYBR Green staining and FCM (see below) and then diluted to a final concentration of 1 × 10^6^–1 × 10^7^ bacteria per ml in 45 ml of 1x PBS. Seawater samples were used directly without wash or dilution.

UV irradiation was performed with a collimated beam apparatus with two 150 W low pressure (LP) UV lamps (BestUV, Hazerswoude, Netherlands), according to recommendations by Bolton et al. (2015). Samples (45 ml) were irradiated in a sterile glass petri dish (diameter 6.7 cm, depth 1.28 cm) with magnetic stirring at 150 rpm (IKA Color Squid, Sigma Aldrich, United States). Calibration was performed with a radiometer (BestUV, Hazerswoude, Netherlands) at 254 nm. The distance between the UV lamp and the sample surface was 83.4 cm. Exposure times for the target fluences were calculated according to setup parameters (Bolton et al., 2015). Cells were exposed to UV irradiation for 2 min 20 s (25 mJ/cm^2^), 4 min 40 s (50 mJ/cm^2^), 7 min (75 mJ/cm^2^), 9 min 20 s (100 mJ/cm^2^), or 18 min 40 s (200 mJ/cm^2^).

For heat treatments, samples were incubated at 55°C for 15, 30, or 45 min in a water bath (GD100, Grant instruments, United Kingdom), with regular manual mixing every 5 min. The PBS buffer used for bacterial dilutions was prewarmed to 55°C, to reach target temperature instantly.

Bacterial inactivation with various UV doses and heat inactivation of different lengths of time were compared to control samples; two untreated samples [with and without L-homopropargylglycine (HPG)], one dead control (dead formalin; DF) where the cells were killed with formaldehyde fixation (4% final concentration, 24 h before the experiment) and incubated with HPG, and another dead control (dead temperature; DT) where the cells were killed by heat treatment at 80°C for 3 min and incubated with HPG.

All samples were finally divided into four replicates of 10 ml, for later addition of HPG after 0, 6, 12, and 24 h of incubation for *E. coli*, and after 0, 6, 24, and 48 h of incubation for natural seawater samples. Incubation time was selected to observe direct and long-term effect of UV irradiation and heat on protein production and possible recovery effects. HPG 15 μM was added, followed by a 3 h of incubation at 37°C and 200 rpm (KS-10, Edmund Bühler, Germany) for *E. coli* and 6 h of incubation at 15°C and 200 rpm (KS-10, Edmund Bühler, Germany) for natural seawater, to allow incorporation of HPG during protein synthesis.

All samples were analyzed with BONCAT, LIVE/DEAD^®^ BacLight™ staining, and plate counts. The BONCAT samples (1 ml) were fixed with filtered formaldehyde (4% final concentration) and kept at 4°C until further analysis. The LIVE/DEAD samples were diluted and stained. Plate counts of *E. coli* and natural seawater bacteria were carried out by spreading 0.1 ml of diluted bacteria on tryptic soy agar (Difco, Becton Dickinson, United States) and 0.1 ml of natural seawater sample on marine agar (Difco, Becton Dickinson, United States), respectively. Plates were incubated for 48 h at 37°C for *E. coli* and 72 h at 15°C for natural samples.

### Bacterial Vitality: Click Chemistry and LIVE/DEAD^®^ BacLight™

Alexa Fluor 647 (AF647) in combination with SYBR Green DNA staining was used for all samples. Fixed samples (volume of ∼1.1 ml) were permeabilized using 1 ml 50% ethanol followed by 3 min of incubation at RT. Ethanol was removed after centrifugation 5 min at 16,000 × *g*. The subsequent incubation in 80 and 96% ethanol followed identical procedures. Samples were resuspended in 1 ml 1x PBS before the click reaction. First, a premix containing 5.75 μl of 20 mM CuSO_4_ (Jena Bioscience, Germany), 11.5 μl of 50 mM Tris-[(1-hydroxypropyl-1*H*-1,2,3-triazol-4-yl) methyl] amine (THPTA) (Click Chemistry Tools, United States), and 0.3 μl of 10 mM AF647 dye (Click Chemistry Tools, United States) was incubated at RT in the dark for 3 min. Second, 57 μl of sodium ascorbate and 57 μl of aminoguanidine hydrochloride (Sigma Aldrich, United States) were added to each sample at a final concentration of 5 mM each. Finally, 17.5 μl of the premix was added to each sample. The samples were gently mixed by inverting the tubes before incubation at RT for 30 min in the dark. After incubation, samples were washed 3 times with 1x PBS buffer by centrifugation at 16,000 × *g*. Following the click reaction, samples were first diluted 10 times in a FCM tube with 1x PBS for *E. coli* or Tris-EDTA buffer (TE) for seawater, then double stained with 10 μl of 100X SYBR Green (final volume 1 ml) (Thermo Fisher scientific, United States), and incubated for 10 min in the dark prior to FCM analysis.

Bacterial vitality was also assessed with the LIVE/DEAD^®^ BacLight™ kit (Thermo Fisher Scientific, United States). The staining protocol followed the manufacturer recommendations. For all samples, 1.5 μl of PI (20 nM in DMSO) and 1.5 μl of SYTO9 (3.34 mM in DMSO) were added to samples diluted 10-fold (1 ml in 1x PBS) and incubated 15 min in the dark before FCM analysis. SYBR Green was used instead of SYTO9 for seawater samples.

### Effect of Lethal Fixative Agents for Dead Control on BONCAT Signal

To evaluate the use of fixatives and possible unspecific background of BONCAT staining, we tested four different fixatives on five different bacteria and seawater. The samples analyzed were seawater samples and cell suspensions of *E. coli*, *A. salmonicida*, *L. anguillarum*, *Y. ruckeri*, and *B. cereus* in 1x PBS. Cell death was obtained by formaldehyde fixation overnight at 4°C (4% final), glutaraldehyde fixation overnight at 4°C (2.5% final), 70% isopropanol 30 min at RT, and 7% acetic acid 25 min at 30°C. A live positive control (with HPG) and a live negative control (without HPG) were included for comparison. Each sample was then incubated with 15 μM HPG for 3 h (6 h for seawater) and 200 rpm mixing (KS-10, Edmund Bühler, Germany) at each bacterium’s respective growth temperature. Samples were subsequently fixed with formaldehyde (4% final) and stored at 4°C before continuing the click chemistry reaction.

### Flow Cytometry

Initial bacterial cultures were counted by FCM after staining with SYBR Green I according to Marie and coworkers (Marie et al., 1999). In short, the cultures were diluted 1,000 times in 1x PBS (1 ml final volume), stained with 10 μl of 100X SYBR Green I (Thermo Fisher Scientific, United States), and incubated 10 min in the dark before FCM analysis. FCM analysis was carried out with an Attune NxT Acoustic Focusing Cytometer (Thermo Fisher scientific, United States) containing a violet laser 405 nm (50 mW), a blue laser 488 nm (50 mW), and a red laser 638 nm (100 mW). Instrument calibration was performed with Attune performance tracking beads (2.4 and 3.2 μm) (Thermo Fisher, United States). The following detectors were used for fluorescence detection: BL1 (530/30) for SYBR Green and SYTO9; BL3 (695/40) for PI; and RL1 (670/14) for AF647. Trigger was set at 2,000 on BL1 (SYBR Green and SYTO9). Compensation was performed for PI/SYTO9 according to the manufacturer’s recommendations. Voltages were optimized for each detector. Between 500 and 10,000 cells were analyzed at a flow rate of 25 μl min^–1^. LIVE/DEAD^®^ BacLight™ gating was performed as instructed by the manufacturer with live (intact membrane, SYTO9 fluorescence), dead (totally permeabilized membrane, PI fluorescence), and damaged (damaged membranes, SYTO9/PI fluorescence) cells. For BONCAT, cells were selected from SYBR Green or side scatter plots and back gated onto SYBR Green or AF647 plots (Lindivat et al., 2020). BONCAT-positive and BONCAT-negative gates were used to determine activity of marine microbes as described previously (Lindivat et al., 2020).

### Statistical Analysis

Experiments were carried out in triplicates with individual triplicates for each sample. Paired *t*-test analyses were carried out to determine significant differences between controls and treated samples.

## Results

### BONCAT Combined With DNA Staining as a Vitality Indicator

**FIGURE 2:**
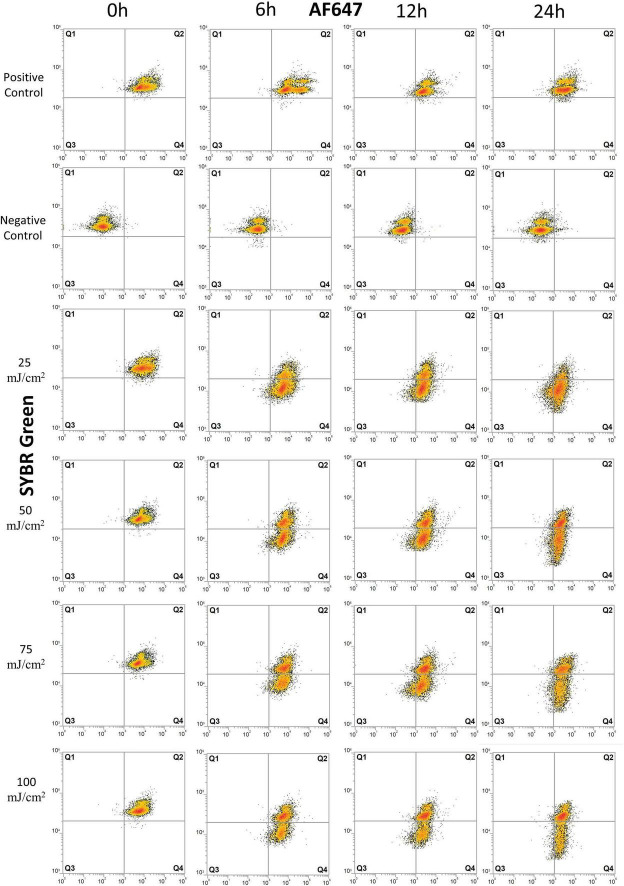
Flow cytometry dot plots of *E. coli* cells treated with UV irradiation. Effects of UV doses of 25, 50, 75, and 100 mJ/m^2^ on *E. coli* were monitored for degradation of DNA and for protein production at 0, 6, 12, and 24 h after treatment. Cells were stained with SYBR Green for DNA detection and AF647 for BONCAT. Between 5,000 and 10,000 cells were analyzed in each dot plot. Quadrant gates were designed from positive and negative HPG control with Q1: dead cells, the presence of intact DNA, negative BONCAT activity; Q2: live cells, the presence of intact DNA, positive BONCAT activity; Q3: dead cells, damaged DNA, negative BONCAT activity; and Q4: damaged cells with damaged DNA and positive BONCAT activity. The results of UV dose of 200 mJ/m^2^ are not included as they present similar pattern as lower UV doses.

**FIGURE 3:**
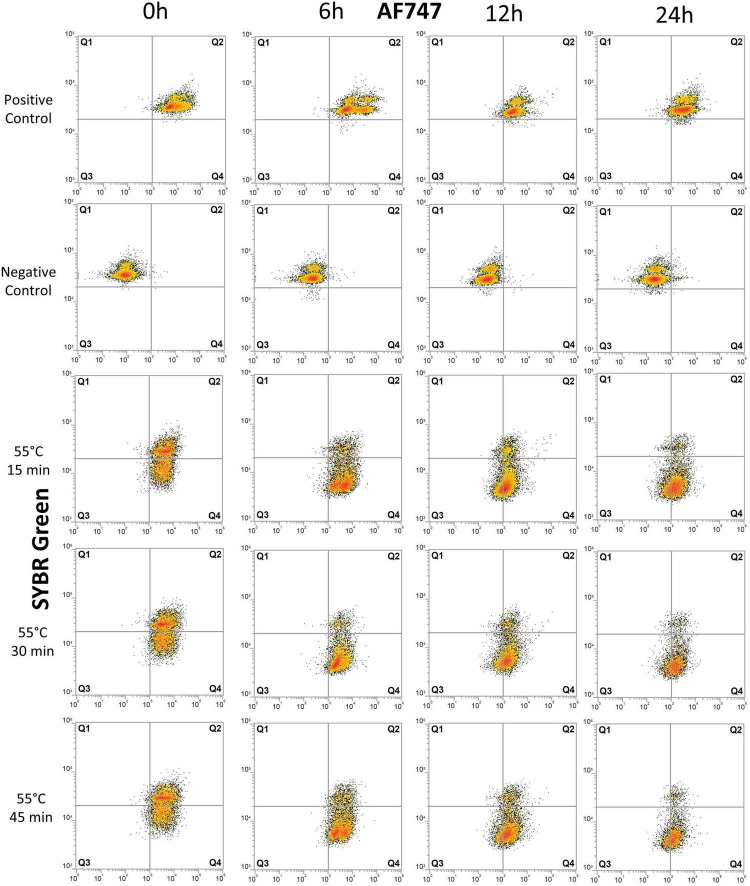
Flow cytometry dot plots of *E. coli* cells treated with heat. The effects of exposure of *E. coli* cells to 55°C for 15, 30, and 45 min were monitored for degradation of DNA and for protein production at 0, 6, 12, and 24 h after treatment. The control samples are the same as those used in the UV experiment (Figure 2). Cells were stained with SYBR Green for DNA detection and AF647 for BONCAT. Between 5,000 and 10,000 cells were analyzed in each dot plot. Gates are identical to those used in Figure 2.

Results from the vitality assessment with BONCAT-FCM are shown in Figures 2, 3 and in Supplementary Table 1 (replicate experiments gave similar results and are not presented).

Flow cytometry analysis was carried out on all treated samples (UV irradiation and heat treatment) and controls (with or without HPG). In addition to BONCAT, all samples were double-stained with SYBR Green. With this combination, the cells could be separated into quadrant gates (Q1–Q4) as described in Table 1.

**TABLE 1:** Overview of bacterial vitality states with BONCAT–SYBR Green.

Quadrant	SYBR Green	BONCAT	Physiological characteristics	Vitality
Q1	+	−	Intact DNA, no protein synthesis	Inactive
Q2	+	+	Intact DNA, protein synthesis	Live
Q3	−	−	Damaged DNA, no protein synthesis	Inactive
Q4	−	+	Damaged DNA, protein synthesis	Damaged

*The different quadrants were obtained from FCM plots. The presence of DNA was detected with SYBR Green and BONCAT with AF647. Gates were determined from controls (positive and negative HPG).*

The positive controls for *E. coli* showed a normal protein synthesis activity throughout the experiment, as demonstrated with 84–99% of active cell in Q2 (Figure 2 and Supplementary Table 1). UV irradiation did not severely affect cells at 0 h as populations remained in Q2, similar to the positive control, but with a small decrease in SYBR Green intensity toward Q4 (Figure 2 and Supplementary Table 1).

Effects of UV irradiation can be observed at 6 h with a decrease in fraction of live cells (Q2) and increase in damaged cells (Q4) (Figure 2 and Supplementary Table 1). The fraction of inactive cells (Q1+Q3 = 0.9–2.7%, Supplementary Table 1) decreased, and we interpret this as a transient response to the transfer to PBS buffer and stable incubation conditions. Protein synthesis remained relatively high after 12–24 h with 26–60% live cells (Q2) and 34–60% of damaged cells (Q4). The percentage of inactive cells (Q1+Q3) varied from < 2% at 6 h to 14% at 24 h, demonstrating that cells maintain protein synthesis for a long period after UV irradiation at UV doses up to 100 mJ/cm^2^. The exception to this is for UV dose of 200 mJ/cm^2^ where 42 and 67% of the cells (Q1+Q3) had no protein synthesis after 6 and 24 h, respectively. UV-treated samples were significantly different from untreated controls (*p* < 0.05).

Heat treatment considerably affected cells immediately after treatment with 20–28% of damaged cell (Q4) (Figure 3 and Supplementary Table 1). The proportion of inactive cells (Q1+Q3) increased from 2–3% at 6 h to 19–27% at 12 h. At 24 h, 59–68% of cells are severely damaged (Q4) and 24–33% are inactive with no BONCAT activity (Q3). Heat-treated samples were significantly different from untreated control samples (*p* < 0.06). In the heat-inactivated dead control (DT), 80–95% of the cells appeared as inactive (mainly in Q1). Low cell counts and the presence of cell debris causing high background noise in the FCM scatter plots precluded a more detailed analysis.

In general, cell concentration, as determined by FCM after staining with SYBR Green, remained around 1.5 × 10^6^–4.5 × 10^6^ cells/ml during the entire experiment for all untreated and UV-treated samples. However, cell concentration in heat-treated samples decreased compared to original concentration (7 × 10^5^–9 × 10^5^ cells/ml) most probably due to cell degradation.

### Comparison of Vitality Methods: LIVE/DEAD BacLight and Plate Counts

**FIGURE 4:**
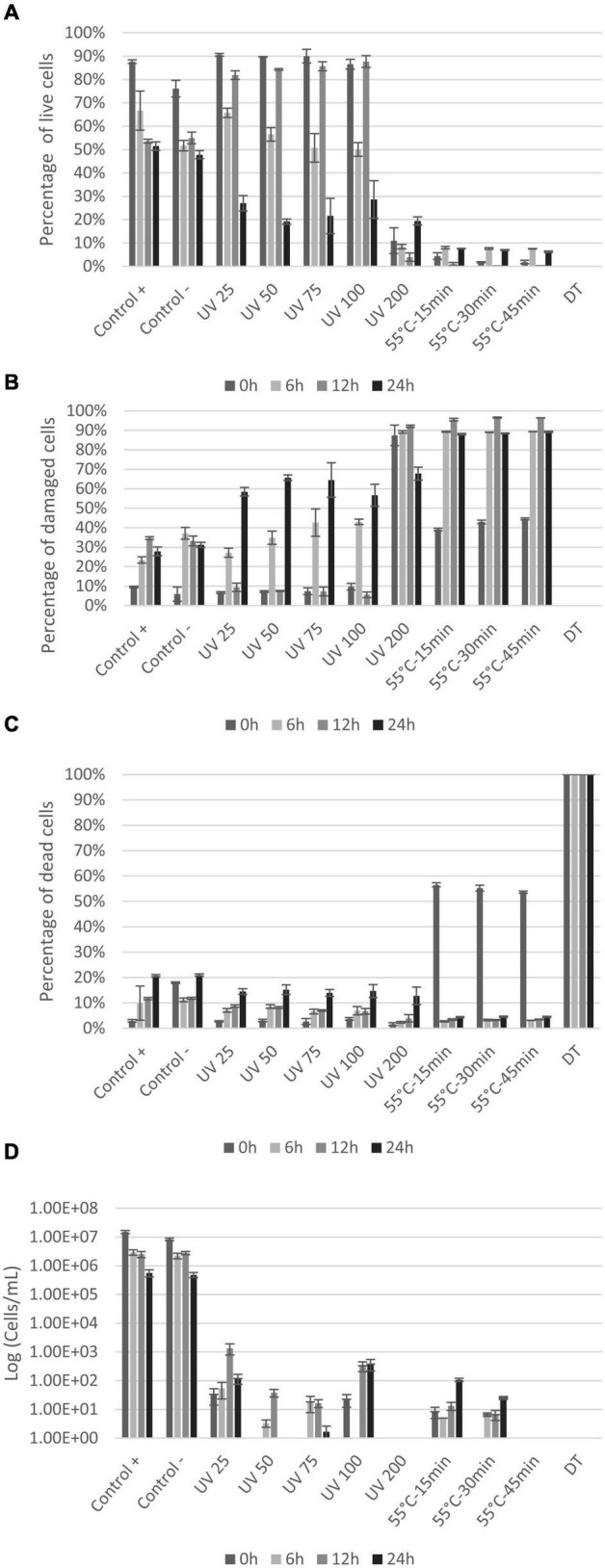
Percentage of live **(A)**, damaged **(B)**, and dead **(C)** *E. coli with* LIVE/DEAD^®^ BacLight™ staining at 0, 6, 12, and 24 h after treatments. Plate counts **(D)** of *E. coli* at 0, 6, 12, and 24 h after treatments. X-axis show the different treatments the cells are exposed to, whereas the y-axis is the percentage of cells **(A–C)** or CFU count in log (cell/ml) **(D)**. Notice that small variations between samples are to be expected since the distinction between live and damaged cells can be difficult to define and will be affected by variations in staining.

Results from the LIVE/DEAD^®^ BacLight™ staining show live, damaged, and dead *E. coli* cells (Figures 4A–C) based on cell membrane permeability. Loss of cell division based on plate counts is shown in Figure 4D. The controls showed that even without any treatment, the fraction of intact cells decreased and permeabilized cells increased, throughout the experiment (from 0 to 24 h) (Figures 4A–C). The several centrifugations to prepare the initial cell suspension can explain the gradual permeabilization over time as cell membrane can be damaged by collisions (Peterson et al., 2012). All UV doses caused permeabilization of cell membranes, with significant decrease in live cells after 6 h (Figure 4A). The percentage of damaged and dead cells increased accordingly (Figures 4B,C). The UV dose of 200 mJ/cm^2^ permeabilized cells immediately (>90% damaged cells at 0 h) with the largest proportion of dead cells at 24 h (Figures 4B,C). Heat treatment significantly permeabilized cells membrane (*p* < 0.004) with only <4.5% of the cells remaining alive immediately after treatment (0 h) (Figure 4A). The percentage of live cells remained below 10% during the entire incubation period (Figure 4A). In the dead control sample (DT), the entire bacterial population was permeabilized and thus detected within the dead gate. Plate counts were compared with BONCAT and LIVE/DEAD^®^ BacLight™ results for each sample (Figure 4D). Initial *E. coli* concentration was around 8 × 10^6^–1 × 10^7^ cells/mL for the controls (positive and negative). UV treatments and heat treatments significantly reduced cell concentration by 4–5 log and 5–6 log, respectively (Figure 4D). The UV dose of 25 mJ/cm^2^ caused a decrease in *E. coli* CFU counts by four orders of magnitude (Figure 4D). Higher doses (50–100 mJ/cm^2^) seem to inactivate all cells but the counts at 12 and 24 h suggest that some cells were not killed but able to revive and grow after the treatment. This regrowth can be explained by cell aggregation and shielding effects (Blatchley et al., 2001) in addition to release of nutrients by cell lysis at high UV doses (Villarino et al., 2003). No growth was observed at any timepoint when 200 mJ/cm^2^ was applied suggesting that all cells were killed.

### Assessment of Treatment for Marine Water Using BONCAT

**FIGURE 5:**
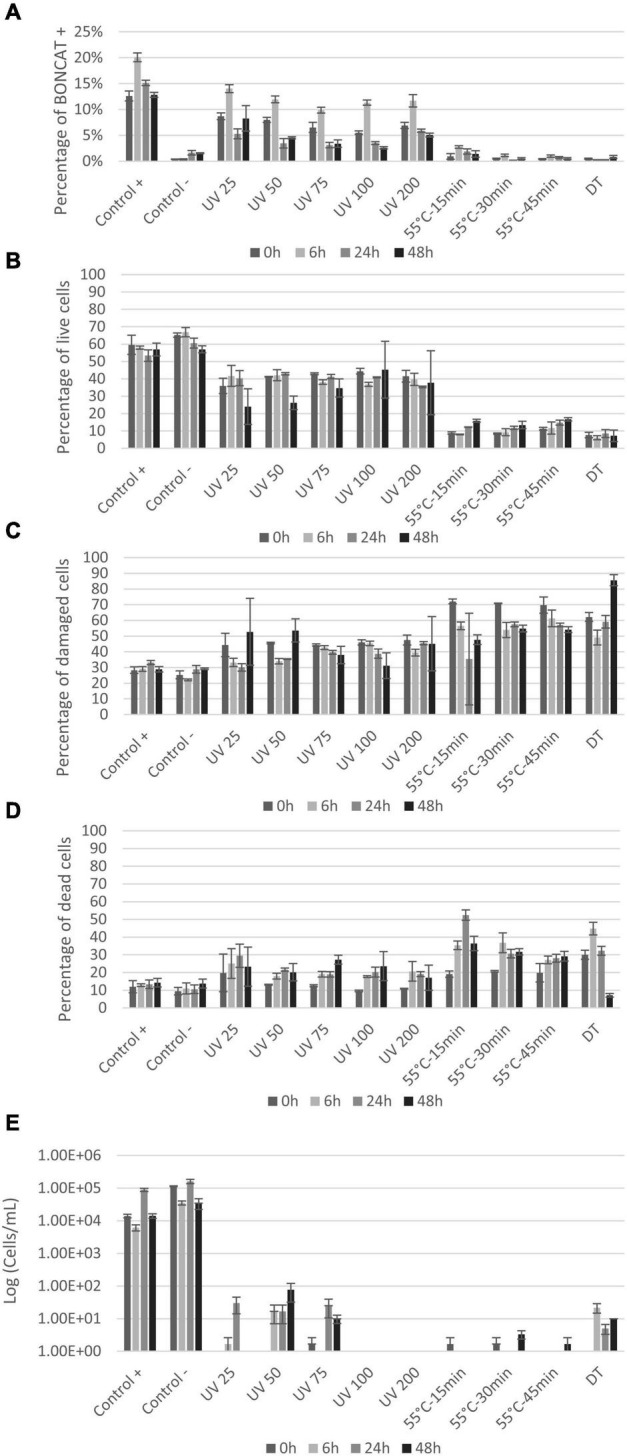
Seawater vitality analysis after treatment: **(A)** Histogram presenting protein synthesis activity of seawater bacterial communities after UV and heat treatments; **(B–D)** percentage of live, damaged, and dead cells by LIVE/DEAD^®^ BacLight™ staining. FCM analysis was carried out on 500–1,000 cells at 0, 6, 24, and 48 h after treatments; **(E)** plate counts from seawater communities at 0, 6, 24, and 48 h after treatments. X-axis shows the different treatments and controls and y-axis represents CFU counts in log (cell/ml).

Natural seawater samples were analyzed with FCM as described previously (Lindivat et al., 2020). For all natural seawater samples, including controls and UV treated samples, an increase in activity was observed after 6 h, which can be explained by the change of environment from natural to laboratory-controlled conditions (Ferguson et al., 1984; Massana et al., 2001). The proportion of bacteria synthetizing proteins was close to 12 ± 2% at the beginning of the experiment (Figure 5A) and increased to 20% after 6 h to reach 12% at 48 h. UV treatments generally decreased this proportion below 5% after 24 h for UV doses of 50, 75, 100, and 200 mJ/cm^2^ (Figure 5A). In general, the proportion of active bacteria decreased with time and increasing UV doses, except for dose of 200 mJ/cm^2^. Heat treatment followed a similar trend with a decrease in the proportion of active cells over time after treatment and with prolonged treatment time. The fraction of active cells was significantly reduced below 2% at all timepoints (Figure 5A). Treated samples with UV and heat were significantly different from control samples (*p* < 0.006 and *p* < 0.03, respectively).

LIVE/DEAD^®^ BacLight™ kit gave somewhat different results compared to BONCAT (Figures 5B–D). For the controls, over 50% of the cells were detected as live, also over time after treatment. The percentage of damaged and dead cells was of 28–32% and 9–14%, respectively (Figures 5C,D). UV treatment caused a permeabilization of cells, but still between 24 and 45% of the cells remained live throughout the experiment (Figure 5B). Simultaneously, the number of damaged cells stayed stable (30–53%), but the proportion of dead cell increased from 9–19% at 0 h to 17–27% at 48 h (Figures 5C,D). The fact that all UV doses gave similar results indicates a similar cell response independently of the UV dose.

For heat treatments, cells were directly damaged (62–72%) with a slight increase in the proportion of dead cells after 6 h of incubation (27–36%) and a stabilization of the different populations at 24 and 48 h (Figure 5D). In comparison, plate counts showed limited growth after all treatments, but regrowth was observed for low UV doses (<75 mJ/cm^2^) (Figure 5E). Some growth was observed in the dead temperature control (DT) meaning the treatment was not fully efficient for that experiment, and results from BONCAT and LIVE/DEAD^®^ BacLight™ kit conformed this observation (Figure 5).

The BONCAT laboratory protocol involves several centrifugation steps and is hence prone to cell loss (Lindivat et al., 2020). In BONCAT-FCM samples, the cell concentration was typically in the order of 10^4^ cells/ml but as low as 10^3^ cells/ml in heat-treated samples. In LIVE/DEAD^®^ BacLight™ samples, the concentration was 10^4^–10^5^ cells/ml. Comparison between treatments was nevertheless possible as we consider fractions and not absolute counts of cells.

### Effect of Lethal Fixative Agents for Dead Control on BONCAT Signal

During the experiments with UV and temperature treatments, we applied a dead control (DF) consisting of cells fixed with formaldehyde. However, fixation of *E. coli* with formaldehyde surprisingly showed a BONCAT+ population comparable to a positive control. To investigate this phenomenon and test whether it is general or specifically related to *E. coli* and/or formaldehyde, we applied the BONCAT method to several different bacterial species and a natural marine bacterial community using different fixatives.

**FIGURE 6:**
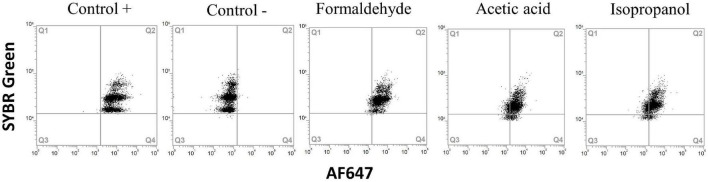
Flow cytometry dot plots of *Yersinia ruckeri.* Cells were treated with acetic acid 7%, 70% isopropanol and fixation with formaldehyde to obtain dead populations. Positive and negative HPG controls were carried out for comparison. All treatments presented a positive protein synthesis activity.

**FIGURE 7:**
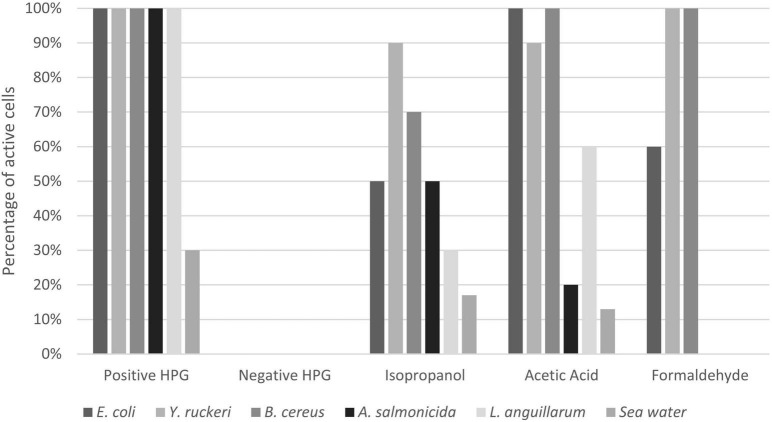
Effect of different fixatives on HPG uptake in different bacteria. Positive HPG is live cells incubated with HPG, whereas negative HPG is the control sample incubated without HPG. The results for the different fixatives are expressed in percentage of active bacteria (number of positive HPG cells). The percentages were obtained by dividing the positive BONCAT cells (Q2) by the total cell number obtained from SYBR Green staining (Q1+Q2).

Fixation with glutaraldehyde gave autofluorescence in the red channel (overlapping with AF647 from the BONCAT staining protocol) and was therefore omitted from the results. Isopropanol resulted in reduced protein synthesis in all samples, but the effect was variable and ranged from 17 to 90% residual activity (Figures 6, 7 and Supplementary Figure 1). Acetic acid and formaldehyde had little or no effect on protein synthesis in *E. coli, Y. ruckeri*, and *B. cereus* while for *A. salmonicida, L. anguillarum* and seawater samples, they resulted in substantial and complete inhibition respectively (Figure 7).

## Discussion

The combination of BONCAT and SYBR Green is a promising double staining technique for the assessment of water quality with respect to microbial activity. BONCAT has been used successfully to sort active vs. non-active bacteria in different environments and subsequently identify them (Hatzenpichler et al., 2016; Couradeau et al., 2019; Reichart et al., 2020). The method offers a new tool to evaluate vitality and activity of microorganisms (Emerson et al., 2017; Hatzenpichler et al., 2020). BONCAT was recently adapted to FCM for routine monitoring with the aim to enhance water microbial quality analysis (Lindivat et al., 2020). BONCAT follows protein synthesis activity *via* incorporation of the amino acid analog HPG. Previous studies of BONCAT showed similar incorporation results compared to radiolabelled [^35^S]methionine and 3H-leucine, reinforcing the capacity of the method to follow protein synthesis in single bacterial cells (Samo et al., 2014; Leizeaga et al., 2017).

A wide range of vitality methods are available for FCM but so far, it is not possible to determine cell death directly (Davey and Guyot, 2020). BONCAT–SYBR Green can be used to distinguish live, damaged, and inactive bacterial cells based on their ability to synthetize proteins and DNA integrity (Figures 2, 3), which are both essential to maintain metabolic activities, cell elongation, and division (Nebe-von-Caron et al., 2000).

The study of *E. coli* vitality with BONCAT after UV irradiation and heat treatment give information about single-cell metabolic activity over time. For the control samples, protein production stayed constant during the experiment. This is expected as stationary phase cells can maintain protein synthesis activity for over 60 h to extend cell longevity (Gefen et al., 2014; Jaishankar and Srivastava, 2017). The effect of UV irradiation was not immediately evident but significant after 6 h and there was apparently no dose-response. The heat treatment had in contrast an immediate impact with an increasing effect according to treatment length (Figure 3). This difference in cell response can be explained by the cellular targets of the treatments (e.g., DNA, proteins, and lipids) and treatment efficiency that will influence metabolic state and cell death (Davey and Guyot, 2020).

UV-C irradiations (254 nm) inhibit replication and transcription by damaging nucleic acids (DNA/RNA) and forming pyrimidine dimers and nucleic lesions (Oguma et al., 2002). UV irradiation as a water treatment technology has been extensively studied with different vitality methods (Hijnen et al., 2006; Safford and Bischel, 2019). UV-C irradiation between 4–500 mJ/cm^–2^ induces a reduction of ATP synthesis, esterase activity, membrane potential, and efflux activity (Villarino et al., 2000, 2003; Schenk et al., 2011). However, cell respiration is not affected by UV irradiation (Villarino et al., 2000; Blatchley et al., 2001; Guo et al., 2019). Villarino et al. (2000) even found residual esterase activity in *E. coli* for 48 h after UV treatment. We observed BONCAT activity in UV-irradiated samples even 24 h after treatment and despite major DNA damage (Figure 2). Villarino et al. analyzed protein synthesis after UV irradiation with the incorporation of [^35^S]methionine and did not detect activity immediately after irradiation with UV dose of 10 and 80 mJ/cm^–2^ (Villarino et al., 2000, 2003). Our results from SYBR Green staining reveal DNA degradation as a main effect after UV irradiation, as shown in Figure 2. DNA degradation is then followed by a decreased but still present protein synthesis, which can be linked to metabolic activities and repair mechanisms for cell recovery. Two types of repair mechanisms are known, the photo repair and the dark repair (Goosen and Moolenaar, 2008). Since our samples were incubated in the dark, it is most likely the action of dark repair systems, such as excision base repair that removes damaged DNA segments (Jungfer et al., 2007).

In comparison, heat treatment had a greater impact on cell integrity and vitality. Heat treatment denatures proteins and liberate membrane components such as lipopolysaccharides but also intracellular enzymes (Tsuchido et al., 1985). We found an accelerated loss of protein synthesis and DNA content linked to treatment time. A similar inactivation has previously been observed for *E. coli* cells treated at 60°C for 30 min (Villarino et al., 2000) and at 85°C (Schenk et al., 2011) maintaining very little esterase activity and had compromised membranes.

Results from BONCAT were compared to well-known methods providing information about other cell functions than protein synthesis. LIVE/DEAD^®^ BacLight™ stains cells with propidium iodide (PI) and SYTO9 to examine membrane permeability. PI enters dead or damaged cells and emits red fluorescence, whereas SYTO9 enters both live and dead cells, binds to DNA and RNA, and emits green fluorescence (Berney et al., 2007). BONCAT and LIVE/DEAD^®^ BacLight™ thus target different cell functions and components and provide complementary information on cell physiology. The UV-treated cells lost their membrane integrity from 12 h, when protein synthesis was at its lowest (Figures 2, 4). This is coherent with previous observations that loss of membrane integrity occurs in the late stage of cell death (Nebe-von-Caron et al., 2000; Berney et al., 2006; Hammes et al., 2011). On the other hand, heat treatment immediately impacted membrane permeability while ribosomes and DNA necessary for protein production were still functional (Figures 3, 4).

BONCAT and LIVE/DEAD^®^ BacLight™ vitality results did not correlate with the results from plate counts examining viability. Similar findings have been reported by others as cells still present activity without cell division (Blatchley et al., 2001; Kramer and Muranyi, 2014). Stressful conditions may induce a viable but non-culturable state (VBNC) that will allow cells to recover or persist despite not being able to divide (Colwell, 2000). VBNC cells maintain their integrity, a low metabolic activity and gene expression (Ayrapetyan and Oliver, 2016). UV irradiation can induce this state, as previously shown for *E. coli* and *P. aeruginosa* (Zhang et al., 2015). Zhang showed that cells were able to resuscitate after low-dose UV irradiation (25 mJ/cm^2^) (Zhang et al., 2015). Several studies have shown that VBNC cells that could not be detected with plate counts still exhibit physiological activity, demonstrated with enzyme activity with substrate CFDA (fluorescein diacetate derivate), membrane potential (DIBAC_4_), and ATP assays (Berney et al., 2008; Kramer and Muranyi, 2014). We also observed similar results as cells were not able to divide on media but still maintained protein synthesis activity and also membrane integrity. Altogether, the three methods evaluate different aspects of cell physiology and provide information on vitality that are necessary to evaluate cell death. As cell death follows a pattern of disfunction ranging from loss of metabolic activity to membrane integrity (Davey, 2011), the three methods are complementary.

For environmental samples, the protein synthesis in general was low which is consistent with previous observation of marine samples analyzed with BONCAT (Lindivat et al., 2020). Marine bacteria display different levels of activity (e.g., inactive, slow growing, starving, dormant, active, and death), but inactivity does not necessarily mean dead (and similarly live does not always mean active) (Del Giorgio and Gasol, 2008). The reduction in protein synthesis related to increasing UV dose is consistent with results by Penru et al. (2013) that observed decreased cellular ATP concentration after irradiation.

For membrane permeability, we applied SYBR Green instead of SYTO9 as it is more used for environmental microorganisms (Marie et al., 1999; Berney et al., 2007) and distinction of live or dead cells was difficult to interpret as described previously (Shi et al., 2007). LIVE/DEAD^®^ BacLight™ staining with PI/SYBR Green showed a higher proportion (50–60%) of live cells than BONCAT (12–20%) for untreated samples. However, BONCAT and PI/SYBR Green showed a similar tendency for heat-treated cells where they lost metabolic activity and membrane activity immediately. Plate counts showed that both treatments efficiently reduced cell concentration. However, as cultivation is not a reliable method to observe a total heterotrophic bacterial population, it was not possible to capture the whole microbial population compared to BONCAT and PI/SYBR Green (Allen et al., 2004).

The cells that were fixed with formaldehyde, even for more than 24 h, showed positive protein synthesis when incubated with HPG (Figures 6, 7). One hypothesis to explain our results is that HPG, if not incorporated, somehow is trapped inside the cells during fixation and protein crosslinking, a process that take more than 24 h (Kiernan, 2000; Metz et al., 2004; Kamps et al., 2019). The observations that incubation with higher HPG concentration (25 vs. 5 μM tested with *E. coli* and *Y. ruckeri*) gave an increase in fluorescence and that adding methionine as a competitive amino acid to HPG when incubating *Y. ruckeri* and *A. salmonicida* did not eliminate the signal (data not shown) supports this hypothesis. There is to our knowledge no coherence (e.g., cell wall type, capsule formation) between the bacteria tested that may explain why they appear to behave differently. A more complete and specific study is necessary to verify this observation, and the possibility of other hypothesis should also be taken into account.

The observation that different fixatives had an inconsistent and incomplete effect on apparent protein synthesis in different bacteria concerns the validity of the BONCAT method (Figure 7). We have no reason to believe that inactive and truly dead cells retain or absorb HPG to yield false positives, and the effect of heat on *E. coli* shown in Figure 3 supports this view. The significance of the phenomenon is, however, difficult to evaluate, for long incubation times and very active cells the relative amount of “false uptake” may be small, while for short incubations and less active cells, it may be important. Assuming that the false uptake comes to saturation after some time, it should be possible to correct for it by subtracting time zero blanks. For the results in this study, this means that the populations (i.e., dot plots) shown in Figures 2, 3 should have been further to the left but we would then have moved the quadrant gates accordingly and our interpretation and conclusions would not have been affected. Formaldehyde seems to be efficient for marine bacteria, and the seawater experiment shown in Figure 6A should hence be valid.

One advantage with BONCAT is that all proteins synthesized within cells are analyzed, compared to assays targeting specific elements such as enzyme activity with esterase substrates and respiration with CTC (Braissant et al., 2020). Combination of BONCAT with FCM reduced analysis time compared to microscopy (Lindivat et al., 2020). The method can detect, enumerate, and differentiate bacterial cells affected by UV and heat treatments. Compared to other vitality stains such as DNA stains or substrates that only requires incubation with the sample, BONCAT has many steps that are not compatible for routine analysis of microbial water quality. In addition, regulations for microbial water quality monitoring, e.g., for drinking water or ballast water, use culture-based techniques or PCR detection focusing on reproduction and detection of DNA instead of vitality (Figueras and Borrego, 2010; First and Drake, 2013). However, BONCAT has the capacity to determine microbial vitality for the development of water disinfection methods.

BONCAT is a promising method to determine vitality of bacterial cells after UV and heat treatment. BONCAT is complementary to other vitality staining methods such as LIVE/DEAD^®^ BacLight™ and culturability. With proper calibration and incubation parameters, the method can be used to evaluate bacterial vitality in cultures and in natural samples.

## Data Availability Statement

The raw data supporting the conclusions of this article will be made available by the authors, without undue reservation.

## Author Contributions

IAH, AL, OKH-E, and GB initiated and supervised the study. ML performed the experimental part. ML wrote the manuscript, with significant inputs from GB, IAH, AL, and OKH-E. All authors contributed to the article and approved the submitted version.

## Conflict of Interest

The authors declare that the research was conducted in the absence of any commercial or financial relationships that could be construed as a potential conflict of interest.

## Publisher’s Note

All claims expressed in this article are solely those of the authors and do not necessarily represent those of their affiliated organizations, or those of the publisher, the editors and the reviewers. Any product that may be evaluated in this article, or claim that may be made by its manufacturer, is not guaranteed or endorsed by the publisher.

## Supplementary Material

Supplementary Figure 1 Histograms of protein synthesis activity with BONCAT (AF647/RL1) for *Aeromonas salmonicida* (AS) and *Yersinia ruckeri* (YR). Cells were fixed with formaldehyde and incubated with or without 15 μM HPG for 3 h. FCM analysis was realized with SYBR Green/AF647 on 5,000–10,000 cells.

Supplementary Table 1 Overview of the percentage of protein synthesis activity and intact DNA in *Escherichia coli* cells over time after treatment with UV irradiation and with heat. Calculations were carried out from BONCAT-FCM results. Quadrants were used as described in Table 1. FCM analysis was realized with SYBR Green/AF647 on 5,000–10,000 cells at 0, 6, 12, and 24 h after treatments.
