# Supplementary figures and images for: Flow Cytometric Analysis of Bacterial Protein Synthesis: Monitoring Vitality After Water Treatment
Source: Front Microbiol. 2021 Dec 10;12:772651. doi: 10.3389/fmicb.2021.772651 (PMC8702973; doi:10.3389/fmicb.2021.772651)

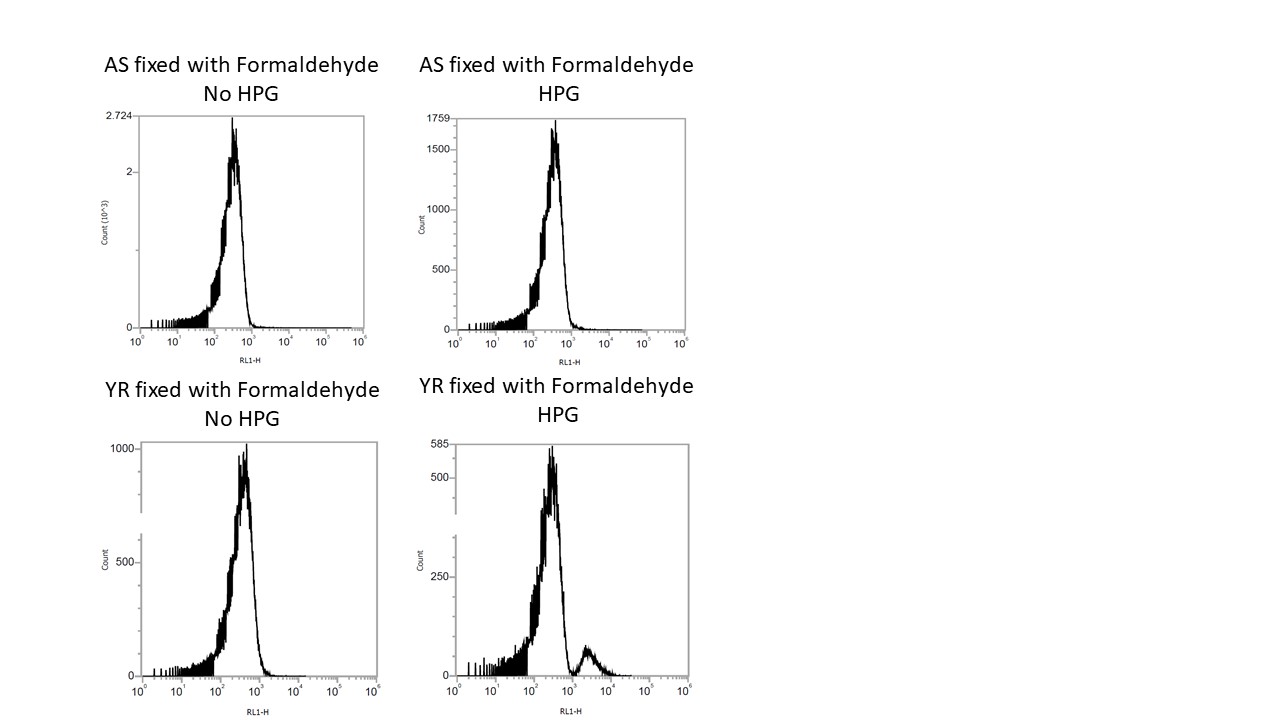

Supplement: Supplementary Figure 1 — Histograms of protein synthesis activity with BONCAT (AF647/RL1) for Aeromonas salmonicida (AS) and Yersinia ruckeri (YR). Cells were fixed with formaldehyde and incubated with or without 15 μM HPG for 3 h. FCM analysis was realized with SYBR Green/AF647 on 5,000–10,000 cells. [file Image_1.JPEG]
