# Supplementary material for: Flow Cytometric Analysis of Bacterial Protein Synthesis: Monitoring Vitality After Water Treatment
Source: Front Microbiol. 2021 Dec 10;12:772651. doi: 10.3389/fmicb.2021.772651 (PMC8702973; doi:10.3389/fmicb.2021.772651)
Supplement: Supplementary Table 1 — Overview of the percentage of protein synthesis activity and intact DNA in Escherichia coli cells over time after treatment with UV irradiation and with heat. Calculations were carried out from BONCAT-FCM results. Quadrants were used as described in Table 1. FCM analysis was realized with SYBR Green/AF647 on 5,000–10,000 cells at 0, 6, 12, and 24 h after treatments. [file Table_1.DOCX]

|  | 0h | | | | 6h | | | | 12h | | | | 24h | | | |
| --- | --- | --- | --- | --- | --- | --- | --- | --- | --- | --- | --- | --- | --- | --- | --- | --- |
| Sample | **Q1** | **Q2** | **Q3** | **Q4** | **Q1** | **Q2** | **Q3** | **Q4** | **Q1** | **Q2** | **Q3** | **Q4** | **Q1** | **Q2** | **Q3** | **Q4** |
| Control HPG + | 12.2 ± 2.56 | 84.4 ± 4.67 | 0.23 ± 0.40 | 0.20 ± 0.35 | 0.28 ± 0.05 | 99.5 ± 0.10 | 0 ± 0.01 | 0.18 ± 0.07 | 3.20 ± 0.58 | 96.4 ± 0.57 | 0.07 ± 0.03 | 0.33 ± 0.05 | 4.78 ± 1.48 | 94.1 ± 1.40 | 0.07 ± 0.02 | 1.05 ± 0.11 |
| Control HPG - | 98.2 ± 1.05 | 1.78 ± 1.05 | 0 ± 0 | 0 ± 0 | 99.0 ± 0.69 | 0.77 ± 0.63 | 0.20 ± 0.08 | 0 ± 0 | 99.8 ± 0.10 | 0.08 ± 0.06 | 0.15 ± 0.05 | 0.01 ± 0.01 | 94.2 ± 3.14 | 4.64 ± 2.94 | 1.10 ± 0.22 | 0.04 ± 0.03 |
| DF | 14.7 ± 2.30 | 85.3 ± 2.30 | 0 ± 0 | 0 ± 0 | 0.88 ± 0.86 | 99.0 ± 0.86 | 0 ± 0 | 0.10 ± 0.04 | 4.18 ± 1.03 | 95.7 ± 1.00 | 0 ± 0 | 0.04 ± 0.03 | 3.78 ± 1.10 | 96.0 ± 1.49 | 0.03 ± 0 | 0.18 ± 0.04 |
| UV 25 | 15.7 ± 1.36 | 78.8 ± 1.80 | 1.30 ± 0.36 | 4.18 ± 0.33 | 0.07 ± 0.03 | 32.7 ± 6.60 | 2.19 ± 0.42 | 65.0 ± 6.14 | 0.65 ± 0.11 | 29.3 ± 6.01 | 8.3 ± 2.61 | 61.8 ± 3.51 | 1.10 ± 0.35 | 26.1 ± 8.90 | 13.0 ± 5.30 | 59.8 ± 4.20 |
| UV 50 | 14.3 ± 3.95 | 82.1 ± 4.24 | 0.77 ± 0.21 | 2.84 ± 0.60 | 0.09 ± 0.03 | 42.1 ± 4.75 | 1.52 ± 0.90 | 56.3 ± 3.90 | 0.56 ± 0.03 | 38.4 ± 4.16 | 6.0 ± 0.7 | 55.1 ± 3.51 | 1.14 ± 1.28 | 36.0 ± 4.35 | 5.90 ± 2.10 | 57.0 ± 6.77 |
| UV 75 | 20.2 ± 10.8 | 77.3 ± 10.1 | 0.79 ± 0.14 | 1.70 ± 0.90 | 2.13 ± 2.80 | 50.9 ± 4.16 | 1.57 ± 0.68 | 45.4 ± 0.68 | 1.17 ± 0.29 | 50.4 ± 6.21 | 7.2 ± 4.0 | 41.3 ± 1.90 | 1.28 ± 0.07 | 45.7 ± 0.26 | 4.56 ± 0.02 | 48.5 ± 0.16 |
| UV 100 | 27.1 ± 1.67 | 71.4 ± 2.20 | 0.64 ± 0.27 | 0.94 ± 0.28 | 0.06 ± 0.04 | 63.0 ± 10.1 | 0.67 ± 0.26 | 36.3 ± 9.83 | 1.92 ± 0.91 | 59.1 ± 2.84 | 5.9 ± 2.7 | 33.1 ± 4.20 | 2.15 ± 1.23 | 60.1 ± 5.93 | 3.60 ± 0.73 | 34.2 ± 6.30 |
| UV 200 | 6.5 ± 2.43 | 93.2 ± 2.64 | 0.02 ± 0.02 | 0.35 ± 0.25 | 32.5 ± 4.30 | 49.7 ± 3.12 | 6.21 ± 5.11 | 5.47 ± 4.33 | 27.9 ± 3.76 | 53.1 ± 6.95 | 10.2 ± 5.38 | 8.81 ± 5.13 | 49.8 ± 3.22 | 27.4 ± 4.40 | 17.4 ± 5.1 | 5.36 ± 1.21 |
| 55°C-15min | 13.4 ± 2.51 | 14.9 ± 2.6 | 43.8 ± 6.93 | 27.9 ± 6.12 | 0.4 ± 0.09 | 14.3 ± 1.15 | 2.09 ± 1.23 | 83.2 ± 0.18 | 2.14 ± 0.58 | 9.29 ± 0.82 | 24.2 ± 5.5 | 64.3 ± 5.74 | 1.23 ± 0.55 | 6.25 ± 0.44 | 23.9 ± 7.36 | 68.1 ± 6.70 |
| 55°C-30min | 12.7 ± 1.52 | 14.1 ± 1.65 | 46.5 ± 5.27 | 26.8 ± 5.14 | 0.31 ± 0.20 | 11.4 ± 4.51 | 2.65 ± 0.47 | 85.7 ± 5.17 | 2.1 ± 0.38 | 9.2 ± 0.77 | 24.2 ± 1.2 | 64.5 ± 2.04 | 1.56 ± 0.28 | 6.81 ± 0.93 | 27.1 ± 7.36 | 64.5 ± 4.50 |
| 55°C-45min | 16.8 ± 1.14 | 13.8 ± 1.10 | 49.6 ± 1.22 | 19.8 ± 1.20 | 0.30 ± 0.04 | 15.5 ± 1.14 | 2.81 ± 0.40 | 81.4 ± 0.92 | 1.5 ± 0.41 | 10.6 ± 1.63 | 17.5 ± 6.2 | 70.4 ± 5.22 | 1.79 ± 0.23 | 6.43 ± 0.39 | 32.8 ± 2.92 | 58.9 ± 2.64 |
